# Photodynamic therapy of DNA mismatch repair-deficient and -proficient tumour cells

**DOI:** 10.1038/sj.bjc.6600218

**Published:** 2002-04-08

**Authors:** V A Schwarz, R Hornung, A Fedier, M K Fehr, H Walt, U Haller, D Fink

**Affiliations:** Department of Obstetrics and Gynaecology, Division of Gynaecology, University Hospital of Zurich, CH-8091 Zurich, Switzerland

**Keywords:** photodynamic therapy, DNA mismatch repair, drug resistance, m-THPC

## Abstract

Loss of DNA mismatch repair is a common finding in hereditary nonpolyposis colon cancer as well as in many types of sporadic human tumours. DNA mismatch repair-deficient cells have been reported to be resistant to many chemotherapeutic agents and to radiotherapy, and to have the potential of rapidly acquiring additional mutations leading to tumour progression. Photodynamic therapy is a new treatment modality using light to activate a photosensitiser that preferentially localises in tumour cells. An oxygen dependent photochemical reaction ensues, resulting in selective tumour necrosis. The effect of loss of DNA mismatch repair activity on the sensitivity to photodynamic therapy was tested using pairs of cell lines proficient or deficient in mismatch repair due to loss of either MLH1 or MSH2 protein function. Cells were incubated with the photosensitiser 5,10,15,20-meta-tetra(hydroxyphenyl)chlorin and exposed to laser light at 652 nm with various optical doses ranging from 0–1 J cm^−2^. Cell survival was assessed using the clonogenic assay. Loss of MLH1 or MSH2 function was not associated with resistance to photodynamic therapy. MCF-7 cells repeatedly treated with photodynamic therapy expressed parental levels of MLH1, MSH2, MSH6, and PMS2. DNA mismatch repair-deficient and -proficient cells showed similar subcellular distributions of meta-tetra(hydroxyphenyl)chlorin as analysed by laser scanning and fluorescence microscopy. Therefore, repeated exposure of tumour cells to photodynamic therapy does not seem to result in loss of DNA mismatch repair, and loss of mismatch repair, in turn, does not seem to contribute to resistance to photodynamic therapy. Our results suggest recommending photodynamic therapy as a strategy for circumventing resistance due to loss of DNA mismatch repair.

*British Journal of Cancer* (2002) **86**, 1130–1135. DOI: 10.1038/sj/bjc/6600218 www.bjcancer.com

© 2002 Cancer Research UK

## 

DNA mismatch repair (MMR) proteins repair mispaired DNA bases and have an important role in maintaining the integrity of the genome (Modrich, 1997). Loss of MMR is the genetic basis for the hereditary nonpolyposis colon cancer syndrome and is a common finding in a variety of sporadic human tumours. Recent studies have documented that loss of MMR is an important mechanism of resistance to a variety of clinically important drugs, including cisplatin (Aebi *et al*, 1996; Fink *et al*, 1996) and the topoisomerase II poisons (Fedier *et al*, 2001). This is due, in part, to the fact that the MMR system can recognise and bind to various types of adducts in DNA. In addition, the genomic instability that accompanies loss of MMR can increase the rate of mutation in coding or regulatory sequences of other genes whose products may play central roles in determining tumour cell sensitivity to drugs. Loss of MMR has been reported in tumour cell lines selected by repeated treatments for resistance to cisplatin, methylating agents and doxorubicin (Aebi *et al*, 1996; Brown *et al*, 1997). Although the reports are controversial there is some evidence that MMR-deficient cells are also resistant to ionising radiation (Fritzell *et al*, 1997; Xu *et al*, 2001). The development of drug resistance during chemotherapy of initially chemosensitive tumours is a frequent problem in clinical oncology, since it may lead to tumour progression and finally to the death of the patient. Thus, finding new treatment modalities effective against MMR-deficient tumour cells is of the utmost clinical importance.

Photodynamic therapy (PDT) is being evaluated as an alternative treatment option for chemotherapy-resistant tumours (Canti *et al*, 1995). PDT uses laser light of appropriate wavelength and energy to activate a systemically applied photosensitiser that concentrates preferentially in malignant tissues. A photochemical reaction ensues, leading to selective tumour necrosis (Dougherty *et al*, 1975). 5,10,15,20-meta-tetra(hydroxyphenyl)chlorin (m-THPC) is a neutral lipophilic second-generation photosensitiser with an absorption maximum at 652 nm. Confocal laser scanning fluorescence microscopy studies of m-THPC *in vitro* have shown a diffuse distribution of the drug in the cytoplasm (McNair *et al*, 1997). Although there is no localisation of the photosensitiser within the nucleus, a low level of potentially mutagenic DNA damage could occur, depending on the photosensitiser, the cellular repair mechanisms and the affected genes (Oleinick and Evans, 1998). Indeed, PDT has been reported to result in DNA lesions such as single-strand breaks and alkali-labile sites, DNA protein crosslink-correlation and DNA degradation as well as in chromosome aberrations (Evans *et al*, 1997; Oleinick and Evans, 1998). One major advantage of PDT over other treatment modalities is that it can be safely repeated several times (Hornung *et al*, 1998). Moreover, PDT has been reported to be effective in multi-drug resistant cell lines (Canti *et al*, 1995).

In the current study, we sought to determine whether loss of MMR affects the sensitivity of tumour cells to PDT. We report here that loss of MMR does not contribute to resistance to PDT and that repeated exposure of cells to PDT in turn, does not result in loss of MMR, meaning that PDT can be recommended for use in tumours deficient in MMR.

## MATERIALS AND METHODS

### Cell lines

The MLH1-deficient human colorectal adenocarcinoma cell line HCT116 was obtained from the American Tissue Culture Collection (ATCC CCL 247, Manassas, VA, USA). Sublines complemented with chromosome 3 (clone HCT116/3-6, identified here as HCT116+ch3) or chromosome 2 (clone HCT116/2−1, identified here as HCT116+ch2) were obtained from Drs CR Boland and M Koi (Koi *et al*, 1994) as were the MSH2-deficient human endometrial adenocarcinoma cell line HEC59 and a subline complemented with chromosome 2 (HEC59+ch2). HCT116 cells contain a hemizygous mutation in MLH1 resulting in a truncated, nonfunctional protein (Boyer *et al*, 1995). Parental HEC59 cells have been shown to contain a frameshift mutation in one allele and a truncating mutation in the second allele of MSH2 and to be deficient in repair activity (Umar *et al*, 1997). The chromosome-complemented sublines HCT116+ch3 and HEC59+ch2 are competent in MMR. Both cell lines were grown in Iscove's modified Dulbecco's medium (Life Technologies, Basel, Switzerland) supplemented with 2 mM L-glutamine and 10% heat-inactivated foetal bovine serum (GIBCO, Basel, Switzerland). Geneticin (400 μg ml^−1^ for HCT116+ch2 and HCT116+ch3 and 600 μg ml^−1^ for HEC59+ch2) (Life Technologies) was added to medium to maintain the chromosome-complemented lines, but all the experiments were carried out in its absence. The absence and presence of expression of MLH1 in HCT116+ch2 and HCT116+ch3 as well as expression of MSH2 in HEC59 and HEC59+2 were verified by immunoblot analysis (data not shown). The oestrogen dependent human breast cancer cell line MCF-7, proficient in MMR, was cultured in Opti-MEM (GIBCO) supplemented with 10% foetal bovine serum, 25 IE ml^−1^ penicillin (GIBCO) and 25 mg ml^−1^ streptomycin (GIBCO). The cell lines tested negative for contamination with *Mycoplasma* spp and cultured as monolayers in a 95% air/5% CO_2_ atmosphere at 37°C. All cell lines used in the experiments form well-defined individual colonies when seeded sparsely on standard tissue culture plates.

### Reagents

The porphyrin-based photosensitiser 5,10,15,20-meta-tetra (hydroxyphenyl)chlorin (m-THPC, Foscan®) with a molecular weight of 680 Daltons was provided by SCOTIA Pharmaceuticals Ltd (Stirling, UK). This second-generation photosensitiser has recently been approved by the European Medicine Evaluation Agency for treatment of head and neck cancer. The absorption maximum of m-THPC is at 652 nm. m-THPC was dissolved in a recommended solution of ethanol : polyethylene-glycol : water (20 : 30 : 50) to a concentration of 5 mg per 10 ml, and stored at 4°C in the dark. 6-Thioguanine was purchased from Sigma (Buchs, Switzerland) and dissolved immediately before use in 0.9% saline.

### PDT experiments

PDT experiments were carried out under dimmed room light as follows. Cells growing in log phase were harvested with EDTA-trypsin and washed with phosphate-buffered saline (PBS). Either 2000 MLH1-proficient or -deficient cells (HCT116), or 10 000 MSH2-proficient or -deficient cells (HEC59) from a single-cell suspension were seeded into 60 mm tissue culture dishes. After 24 h, 0.1 μg ml^−1^ m-THPC diluted in tissue culture medium was added to the dishes and cells were incubated with m-THPC for 24 h. Cells not exposed to either m-THPC or laser light were used as controls. Then, cells were exposed to laser light at a wavelength of 652 nm generated by a diode laser (Applied Optronics Corp., South Plainfield, NJ, USA) and an energy of 25 mW as verified by a power meter (Fieldmaster Coherent Inc., Santa Clara, USA). The light was conducted through a laser fibre terminated by a front lens light diffuser to the site of irradiation. The optical dose (J cm^−2^) is defined as the fluence rate (W cm^−2^) multiplied by the exposure time in seconds. Irradiation times for the colorectal and the endometrial cancer cells were 0, 7, 14, 28, 42 or 56 s, resulting in optical doses of 0, 0.125, 0.25, 0.5, 0.75 or 1 J cm^−2^ at a fluence rate of 36 mW cm^−2^. The cells were then washed, fresh medium was added and the cells were allowed to proliferate for 10 days.

### Repetitive PDT exposure of MCF-7 cells

MCF-7 breast cancer cells (10^6^) growing in log phase were seeded into 60 mm tissue culture dishes. After 24 h, m-THPC was added to a final concentration of 0.1 μg ml^−1^ and incubated for 24 h in the dark, followed by illumination for 5 min with a fluence rate of 25 mW cm^−2^ resulting in an optical dose of 2.12 J cm^−2^. This PDT dose resulted in a survival fraction of approximately 10^−4^. Surviving cells were allowed to proliferate to a density of 10^6^ cells, and the experiment was repeated for a total of five times. The repetition was restricted to five times due to the fact that from the clinician's point of view it is very unlikely that recurrent tumours will be treated more than five times with the same treatment modality.

### Clonogenic assay

Cell survival was assessed by means of the clonogenic assay 10 days after PDT exposure. Cells were fixed with 25% acetic acid in ethanol and stained with Giemsa. Colonies of at least 50 cells were scored visually. Each experiment was performed at least three times using triplicate cultures for each optical dose. Cell survival was expressed as a fraction of treated to untreated cells (survival fraction, SF) at a relative plating efficiency of 0.5% for the HCT116 and 0.3% for the HEC59 cell lines.

### Immunoblot analysis

Parental and PDT-treated MCF-7 cells were harvested as described before and lysed on ice in 150 mM NaCl containing 5 mM EDTA, 1% Triton X-100, 10 mM Tris/HCl (pH 7.4), 5 mM dithiothreitol, 0.1 mM phenylmethylsulphonyl fluoride and 1 μg ml^−1^ aprotinin, followed by centrifugation at 14 000 **g** for 20 min at 4°C. The protein amount was determined using the Bio-Rad protein assay dye (Bio-Rad, Glattbrugg, Switzerland). After centrifugation, 50 μg protein were denaturated by boiling at 95°C for 5 min in an equal volume of 130 mM Tris/HCl (pH 6.8) containing 20% glycerol, 4.6% sodium dodecyl sulphate (SDS) and 0.02% bromophenol blue. The proteins were separated using SDS–PAGE on a 7.5% gel followed by blotting onto a polyvinylidene difluoride membrane (Amersham Pharmacia Biotech, Buckinghamshire, UK). The MMR proteins were detected using anti-MLH1 (clone G168-728, PharMingen, Heidelberg, Germany), anti-MSH2 (clone FE11, Calbiochem, Lucerne, Switzerland), anti-MSH6 (provided by Dr J Jiricny) and anti-PMS2 (clone A16-4, PharMingen). β-tubulin was used as a loading control. The monoclonal antibody G168-728 was generated with a full-length MLH1 protein, whereas clone FE11 is a mouse monoclonal antibody generated with a carboxyl-terminal fragment of the MSH2 protein. The polyclonal anti-MSH6 is directed against the full-length protein. The monoclonal antibody A16-4 was prepared with a carboxyl-terminal fragment of the PMS2 protein. After washing the blots, horseradish peroxidase-conjugated antimouse antibody (Amersham Life Science, Buckinghamshire, UK) was added, and the complexes were visualised by enhanced chemiluminescence (Amersham Life Science).

### Subcellular drug distribution and kinetics of drug uptake

A confocal laser scanning microscope (Leica TCS 4D, Glattbrugg, Switzerland) equipped with an Argon Krypton laser was used to investigate subcellular photosensitiser distribution. HCT116+ch2 and HCT116+ch3 cells were grown on cover slides and incubated for 24 h with 0.1 μg ml^−1^ m-THPC. The dye was excited at 488 nm and fluorescence was detected above 590 nm with a longpass filter. Kinetics of drug uptake were studied using a fluorescence microscope (Leitz DMRBE, Leica, Glattbrugg, Switzerland) equipped with a computer-controlled charge coupled device camera (Photometrics Ltd., Tucson, AZ, USA). A 530 out of 595 nm bandpass filter and a 615 nm longpass filter were used for excitation and for detection of emission, respectively. Five micrographs per slide were taken and two cells per picture were analysed. Fluorescence intensity was quantified (counts per pixel) as a function of the incubation time using IPLab Spectrum software (Scanalytics, Fairfax, VA, USA). Experiments were repeated five times.

### Statistical analysis

Mean±s.d. values were calculated for all data sets. The two-sided paired *t*-test was used to compare the effect of loss of MLH1 or MSH2 on PDT sensitivity. IC_50_-values were calculated by log-linear interpolation. *P*<0.05 was considered to be a statistically significant difference.

## RESULTS

### Clonogenic cell survival after PDT

**Figure 1:**
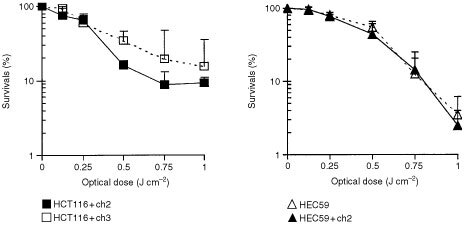
Clonogenic survival curves for PDT for the MLH1-deficient and -proficient colon carcinoma cell lines and the MSH2-deficient and -proficient endometrial carcinoma cell lines. Each point represents the mean±s.d. of at least three experiments performed in triplicate.

A point investigated was whether the sensitivity of tumour cells to PDT is affected by the MMR status. 6-Thioguanine, to which repair-deficient cells were, as expected, 4.2- (IC_50_; HCT116+ch2) and 5.6-fold (IC_50_; HEC59) more resistant than the repair-proficient sublines, was included as a control. Figure 1

shows the survival curves for the MLH1-deficient HCT116+ch2 and the MSH2-deficient HEC59 cells as well as the respective repair-proficient HCT116+ch3 and HEC59+ch2 cell lines after PDT as a function of the optical dose (J cm^−2^). The optical dose required to induce cell death in 50% of all cells (IC_50_) was 0.32±0.03 J cm^−2^ for the HCT116+ch2 and 0.39±0.20 J cm^−2^ for the HCT116+ch3 cells (*P*=0.57). Likewise, MSH2-deficient tumour cells showed no altered sensitivity to PDT. The corresponding IC_50_-values were 0.54±0.06 J cm^−2^ for the MMR-deficient HEC59 and 0.46±0.17 J cm^−2^ for the repair-proficient HEC59+ch2 cells (*P*=0.24). Thus, loss of MMR does not result in resistance to PDT.

### MMR protein expression in MCF-7 cells after repetitive PDT exposure

**Figure 2:**
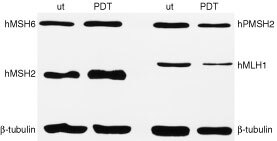
Immunoblot of untreated MCF-7 cells (ut) and MCF-7 cells after five subsequent PDT exposures (PDT). MCF-7 cells repeatedly treated with PDT express parental levels of MLH1, MSH2, MSH6 and PMS2. β-tubulin was used as a loading control.

The question was addressed as to whether repetitive treatments with PDT result in *de novo* loss of expression of MMR proteins in the parental human breast cancer MCF-7 cells. These cells express MLH1, PMS2, MSH2, and MSH6 in amounts that are readily detectable by immunoblot, and they have previously been reported to be sensitive to PDT (Hornung *et al*, 1998). Expression of MMR proteins in MCF-7 cells after five subsequent exposures to PDT and in untreated MCF-7 cells was determined by immunoblot analyses. Figure 2

shows that MCF-7 cells repeatedly treated with PDT express parental levels of MLH1, MSH2, MSH6 and PMS2. The sensitivity of the MCF-7 cells after five PDT treatments was similar to that of MCF-7 cells after a single exposure. Therefore, repeated exposure of tumour cells to PDT does not result in loss of MMR proteins.

### Kinetics of drug uptake and subcellular photosensitiser distribution

**Figure 3:**
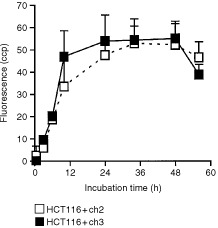
Photosensitiser-mediated fluorescence intensity (counts per pixel, cpp) for MLH1-deficient and -proficient tumour cells shown as a function of incubation time. Each point represents the mean±s.d. of six experiments.

The kinetics of uptake of m-THPC (0.1 μg ml^−1^) were studied in the MMR-deficient HCT116+ch2 and -proficient HCT116+ch3 cells by measuring the photosensitiser-mediated fluorescence intensity (counts per pixel, arbitrary unit) as a function of the incubation time. Figure 3

shows that m-THPC-mediated fluorescence intensity markedly increased within 24 h and reached a plateau with highest fluorescence intensity at 34 h after incubation in MLH1-proficient as well as in MLH1-deficient cells. Thus, m-THPC uptake occurs within 24 h and follows similar kinetics in both cell lines.

**Figure 4:**
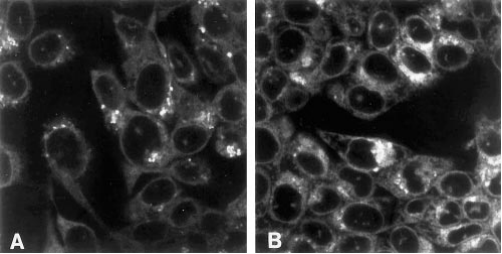
Bright field image of HCT116+ch3 (**A**) and HCT116+ch2 (**B**) after incubation with 0.1 μg ml^−1^ m-THPC for 24 h. After 24 h of incubation there was no detectable fluorescence within the nucleus. The nuclear membrane is distinctly stained in both cell lines.

The subcellular distribution of m-THPC was analysed by confocal laser scanning microscopy. m-THPC-mediated fluorescence intensity in the cytoplasm of HCT116 cells increased within 24 h after administration. At that time, only weak fluorescence intensity was associated with the nuclear membrane. Figure 4

represents a typical example of fluorescence distribution in the HCT116+ch3 (Figure 4A) and HCT116+ch2 (Figure 4B) cell lines after 24 h of m-THPC administration, demonstrating that the fluorescence pattern of HCT116+ch3 was similar to that of HCT116+ch2 cells. Likewise, longer incubation with m-THPC resulted in increased fluorescence intensity associated with membranous structures in the cytoplasm, whereas the nucleus did not show detectable fluorescence at any time after photosensitisation. Thus, drug uptake kinetics and subcellular distributions are very similar in MMR-proficient and -deficient cells.

## DISCUSSION

MLH1 or MSH2-deficient tumour cells have been reported to be resistant to a large number of anticancer drugs (Aebi *et al*, 1997; Fink *et al*, 1998b) and to radiotherapy (Fritzell *et al*, 1997; Xu *et al*, 2001), and as a result, MMR status may be an important resistance factor. Although loss of MMR results in relatively small degrees of resistance, there is evidence that this resistance is nevertheless of substantial clinical significance. The strongest clinical evidence correlating chemotherapy responses with the *in vitro* data has been reported from studies on ovarian and breast cancers. We and others (Brown *et al*, 1997; Fink *et al*, 1998a; Samimi *et al*, 2000) have shown that there is an increase in the number of ovarian tumour cells that score negative for MLH1 expression following platinum-based chemotherapy when compared with untreated tumours. Recently, studies have correlated tumour response and poor disease-free survival with loss of MLH1 expression in breast cancer tumours following anthracycline-based neoadjuvant chemotherapy (Mackay *et al*, 2000). These observations support the concept of *in vivo* enrichment of a subpopulation of MMR-deficient cells in response to treatment, which are more likely to be drug resistant, have a mutator phenotype and consequently, an adverse effect on prognosis. Similarly, it has been reported that low levels of MLH1 and MSH2 in malignant gliomas correlate with resistance to temozolomide, a methylating agent (Friedman *et al*, 1998).

PDT is a relatively new treatment modality for malignant tumours. Selective tumour cell necrosis is induced by a distinct photochemical mechanism. While chemoresistance in MMR-deficient tumours seems to force physicians to surrender an efficient anticancer treatment, the present study suggests that PDT might offer a future treatment option for these patients. So far, it has been reported that PDT does not induce resistance to chemo- or radiotherapy, and that this treatment can be repeated without increasing toxicity and with low probability of inducing resistance to PDT (Sharkey *et al*, 1993; Luna *et al*, 1995; Hornung *et al*, 1998; Singh *et al*, 2001). Our study extends the theoretical advantages to conventional treatment modalities for cancer by demonstrating that loss of MMR does not result in resistance to PDT.

Loss of MMR has been reported in tumour cells selected by repeated treatments with cisplatin, methylating agents or doxorubicin (Aebi *et al*, 1996; Brown *et al*, 1997). Therefore, we analysed by immunoblot the presence of the MMR proteins in MCF-7 cells that survived five subsequent cycles of PDT. As shown in Figure 2, the PDT-treated MCF-7 cells expressed parental levels of MLH1, MSH2, MSH6 and PMS2. Thus, PDT is not only effective against MMR-deficient cells but - unlike some chemotherapeutic agents – it does not result in loss of MMR, allowing standard chemo- or radiotherapy following PDT-mediated tumour treatment.

PDT-induced cell killing is not fully understood and may depend on the photosensitiser and the treatment protocol used. However, the potential of PDT to induce genotoxic damage seems to be relatively low compared with ionising radiation or chemotherapy (Evans *et al*, 1997). This may, in part, be explained as follows. As demonstrated in Figure 4, the cationic and lipophilic photosensitiser m-THPC localises in cellular membranes, mainly in the mitochondria with highly negative electrochemical potential of the inner membrane, and to a lower extent in the nuclear membrane. Singlet oxygen, the major mediator of the PDT-induced photochemical reaction (Henderson and Dougherty, 1992), has a very short diffusion distance of 0.01 μm and a very short lifetime of 0.01 μs (Moan and Berg, 1991). The photochemical reaction may therefore reach only DNA that is located very close to the nuclear membrane (Evans *et al*, 1997). Oxidative damage leading to single-strand breaks and alkali-labile sites, DNA-protein crosslinks and DNA degradation, as well as to chromosome aberrations has been reported (Evans *et al*, 1997; Oleinick and Evans, 1998). These lesions, however, are likely to be relatively easily repaired (McNair *et al*, 1997). Considering that MMR seems not to be affected by PDT, it is reasonable to assume that PDT-induced DNA damage remains of low clinical importance. Indeed, the risk of PDT generating secondary cancer is known to be very small (Moan and Berg, 1992).

In order to ensure that PDT acts similarly in MMR-proficient and -deficient tumour cells, the subcellular photosensitiser distribution and kinetics of drug uptake (i.e. relative values representing changes in drug concentrations) have been estimated using fluorescence microscopy. As demonstrated in Figures 3 and 4, both, drug uptake kinetics and drug distribution, were similar in MMR-proficient and -deficient cells. This finding further supports the idea that PDT-mediated cell killing is fully independent of MMR. Furthermore, the pattern of the subcellular photosensitiser distribution and the kinetics of drug uptake in HCT116 cells are in good agreement with previous findings of this laboratory in MCF-7 and V-79 cells that are known to be proficient in MMR (Hornung *et al*, 1997).

The addition of mitomycin C has been shown to enhance the PDT effect (Nahabedian *et al*, 1988). Recently, it has been reported that loss of MMR is specifically associated with hypersensitivity to mitomycin C (Fiumicino *et al*, 2000). Although mechanistic studies are needed to fully elucidate the biochemical events involved, these findings are interesting because they may suggest a means of selectively eliminating cells that have lost their ability to perform MMR.

In conclusion, our results demonstrate that: (i) PDT is as efficient in MMR-deficient cells as in MMR-proficient cells; (ii) repetitive treatments with PDT do not result in loss of MMR; and (iii) MMR-deficient cells show similar m-THPC distribution and kinetics of drug uptake as cells proficient in MMR. Thus, our results suggest the use of PDT as a strategy for circumventing resistance due to loss of MMR.
